# Supplementary material for: Content-rich biological network constructed by mining PubMed abstracts
Source: BMC Bioinformatics. 2004 Oct 8;5:147. doi: 10.1186/1471-2105-5-147 (PMC528731; doi:10.1186/1471-2105-5-147)
Supplement: Additional File 2 — The original results of the above study (non-essential files are deleted to keep the file size under the limit set by BMC bioinformatics). [file 1471-2105-5-147-S2.bz2 › chilibotAdditionalFile2/dip05/751ID11872715E18134/html/FEPA_TONB.html]

 


 **FEPA** and **TONB** 
  
Found 47 abstracts in PubMed, retrieved 05.  
 

 What does Google say? 
 PDF only 
| .edu only 

---

**Interactive relationship** (e.g. stimulation, inhibition, etc)

**Non-interactive relationship** (e.g. studied together, co-existance, homology, etc.)

- The enterobactin degradation product, dihydroxybenzoylserine DBS was as effective as NE in stimulating the growth of E. coli and mutations in  **fepA**  or  **tonB**  abolished the DBS dependent growth stimulation.  Ref: 12379665 Infect Immun, 2002
- The crystallographic structures of two  **TonB**  dependent receptors FhuA and  **FepA**  have recently been determined.  Ref: 12409204 Biochim Biophys Acta, 2002
- Consistent with this hypothesis, E. coli strains with mutations in ferrienterobactin transport  **fepA**  or  **tonB**  or enterobactin biosynthesis entA did not respond to NE.  Ref: 12379665 Infect Immun, 2002
- We consider a variety of different colicins, including A, B, D, E1, E3, Ia, M and N, that penetrate through the porins OmpF,  **FepA** , BtuB, Cir and FhuA, to subsequently interact with a few targets in the periplasm, including TolA, TolB, TolC and  **TonB** .  Ref: 12423783 Biochimie< MedlineTA>Biochimie, 2002
- DALTV of FecA could be functionally replaced with the core  **TonB**  boxes of FhuA DTITV and  **FepA**  DTIVV.  Ref: 12618451 J Bacteriol, 2003
